# Associations between atypical intracortical myelin content and neuropsychological functions in middle to older aged adults with ASD

**DOI:** 10.1002/brb3.3594

**Published:** 2024-06-07

**Authors:** Jiwandeep S. Kohli, Annika C. Linke, Ian A. Martindale, Molly Wilkinson, Mikaela K. Kinnear, Alan J. Lincoln, Janice Hau, Ian Shryock, Vinton Omaleki, Kalekirstos Alemu, Stephanie Pedrahita, Inna Fishman, Ralph‐Axel Müller, Ruth A. Carper

**Affiliations:** ^1^ Brain Development Imaging Laboratories, Department of Psychology San Diego State University San Diego California USA; ^2^ San Diego Joint Doctoral Program in Clinical Psychology San Diego State University/University of California San Diego California USA; ^3^ California School of Professional Psychology Alliant International University San Diego California USA

**Keywords:** aging, autism spectrum disorder, cortex, myelin, neuroimaging, neuropsychology

## Abstract

**Introduction:**

In vivo myeloarchitectonic mapping based on Magnetic Resonance Imaging (MRI) provides a unique view of gray matter myelin content and offers information complementary to other morphological indices commonly employed in studies of autism spectrum disorder (ASD). The current study sought to determine if intracortical myelin content (MC) and its age‐related trajectories differ between middle aged to older adults with ASD and age‐matched typical comparison participants.

**Methods:**

Data from 30 individuals with ASD and 36 age‐matched typical comparison participants aged 40–70 years were analyzed. Given substantial heterogeneity in both etiology and outcomes in ASD, we utilized both group‐level and subject‐level analysis approaches to test for signs of atypical intracortical MC as estimated by T1w/T2w ratio.

**Results:**

Group‐level analyses showed no significant differences in average T1w/T2w ratio or its associations with age between groups, but revealed significant positive main effects of age bilaterally, with T1w/T2w ratio increasing with age across much of the cortex. In subject‐level analyses, participants were classified into subgroups based on presence or absence of clusters of aberrant T1w/T2w ratio, and lower neuropsychological function was observed in the ASD subgroup with atypically high T1w/T2w ratio in spatially heterogeneous cortical regions. These differences were observed across several neuropsychological domains, including overall intellectual functioning, processing speed, and aspects of executive function.

**Conclusions:**

The group‐level and subject‐level approaches employed here demonstrate the value of examining inter‐individual variability and provide important preliminary insights into relationships between brain structure and cognition in the second half of the lifespan in ASD, suggesting shared factors contributing to atypical intracortical myelin content and poorer cognitive outcomes for a subset of middle aged to older autistic adults. These atypicalities likely reflect diverse histories of neurodevelopmental deficits, and possible compensatory changes, compounded by processes of aging, and may serve as useful markers of vulnerability to further cognitive decline in older adults with ASD.

## Graphical abstract

When examined using subject‐level analyses, a subgroup of middle to older aged autistic adults with atypically high myelin content in spatially heterogeneous cortical regions showed lower neuropsychological function in multiple cognitive domains.

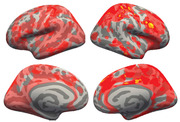

## 1 INTRODUCTION

In vivo myeloarchitectonic mapping based on MRI provides a unique view of gray matter content and offers complementary information to the morphological indices that have been commonly employed in many studies of brain‐behavior relationships (e.g., cortical thickness, surface area, etc.). Intracortical myelin development and remodeling are protracted across the human lifespan, and there is evidence of atypical cortical myelination in some neuropsychiatric disorders (Lake et al., 2017), as well as in age‐related mild cognitive impairment and dementia (Bouhrara et al., 2018). Autism spectrum disorder (ASD) is a neurodevelopmental disorder characterized by social communication deficits and restricted and repetitive behaviors (American Psychiatric Association, 2013) and is a lifelong condition (Robison, 2019), but there is a deficit of knowledge about the neurobiological, cognitive, and behavioral changes that may occur across the later portion of the lifespan in ASD. In young adults with ASD, myelin content (MC) has been shown to be reduced in white matter (Deoni et al., 2015), but patterns of intracortical myelination specifically have not yet been examined in older adults with ASD. With the added risk of demyelination associated with aging, middle to older aged adults with ASD are an important population to examine, as they may be at a particularly heightened risk for alterations in cortical myelination.

### 1.1 Ongoing intracortical myelin development and changes in adulthood

Myelin in the brain is formed by oligodendrocytes, rich in lipids, that wrap processes around axons and facilitate the conduction of electrical impulses. Myelination in the human brain is unique in its protracted maturation, reaching its peak in multimodal association areas of the prefrontal cortex only in middle age. (Sowell et al., 2003) These spatial and temporal patterns established in postmortem studies by the early 20th century neuroanatomists have been replicated using MRI‐derived myelin maps, which further confirm that intracortical myelin maturation is ongoing at least into the fourth decade of life, followed by 20 relatively stable years before declining from the sixth decade (Grydeland et al., 2013). While myelination speeds axonal conduction in white matter beneath the cortex (Hartline & Colman, 2007), it has been suggested that a major function of *intracortical* myelination is to inhibit the formation of aberrant or superfluous connections (Braitenberg, 1962). This hypothesis is supported by a considerable amount of molecular evidence showing that myelin‐related factors inhibit new axonal growth and synapse formation (M. S. Chen et al., 2000; Kapfhammer & Schwab, 1994; McGee et al., 2005; McGee & Strittmatter, 2003; McKerracher et al., 1994). Therefore, regional deficits and atypical degeneration of intracortical myelin may increase susceptibility to aberrant connections, which might in turn underlie cognitive and behavioral symptoms like those seen in developmental conditions such as ASD.

### 1.2 Cognitive and neuroanatomical changes in middle to older aged adults with autism

Based on the available studies of adults ranging in age from 18 to 48 years, it is clear that a large portion of individuals with ASD continue to show some degree of cognitive impairment and require support beyond childhood (Billstedt et al., 2005; Howlin et al., 2004; Szatmari et al., 1989; Wing & Shah, 2000). Additionally, a large literature in typical aging shows a cognitive decline after 50 years of age (Hedden & Gabrieli, 2004). Given the existing deficits that many individuals with ASD have in the first half of the lifespan (Levy & Perry, 2011; Magiati et al., 2014; Steinhausen et al., 2016), older individuals with ASD may experience less resilience to neurocognitive changes associated with aging. A few preliminary studies in middle to older aged adults with ASD have indeed found continuation and potential exacerbation of existing symptomatology along with accelerated neuroanatomical changes, including reduced cortical thickness in brain areas related to social cognition in adults with ASD aged 20–55 years (Scheel et al., 2011) and reduced cerebellar volume in adults aged 18–58 years (Hallahan et al., 2009). Other anatomical studies examining age‐related effects have indicated differential rates of morphological change across adulthood compared to typical controls (Braden & Riecken, 2019; Kohli et al., 2019). Connectivity studies have suggested both functional and anatomical differences underlying motor dysfunction in adults with ASD, including reduced and more variable sensorimotor cortex functional connectivity (Linke et al., 2020) and diminished morphological laterality and u‐fiber connectivity based on diffusion MRI data (Hau et al., 2022).

### 1.3 Links between intracortical myelin content and cognition and neuropsychiatric disorders

Thus far, attempts to link lifespan trajectories of intracortical myelin content to cognitive functioning have been limited, even in typical populations, and associations in neurological disorders have largely been indirect, based on postmortem studies. One study of healthy adults aged 20–83 years showed that better performance stability on a speeded task was correlated with a greater degree of intracortical myelination as measured by T1w/T2w ratio, and the relationship was more prominent with advancing age, suggesting that age‐related cortical demyelination contributes to dysfunctional increase in intraindividual variability in performance (Grydeland et al., 2013). There is also evidence of demyelination in mild cognitive impairment and dementia (Bouhrara et al., 2018).

Notably, aberrant intracortical myelination has been implicated in neuropsychiatric disorders such as schizophrenia and bipolar disorder, where postmortem myelin stains have shown reduced myelin localized to dorsolateral prefrontal cortex compared to controls (Lake et al., 2017). An MRI study of myelin water fraction in young adults with ASD showed widespread reduction of myelin content in cerebral white matter (Deoni et al., 2015). Another MRI study found differential age‐related trajectories of T1w/T2w ratio between typically developing (TD) preschoolers and those with ASD, with increasing MC in early myelinating regions, such as visual, posterior cingulate, and precuneus cortices, observed among TD children but not among children with ASD (B. Chen et al., 2022). The trajectory of intracortical myelination across the lifespan in ASD remains understudied, however, and there have yet to be in vivo investigations into cortical myeloarchitectonics across adulthood in autism.

### 1.4 In vivo study of estimated myelin content and associations with cognition in adults with autism

MRI‐based intracortical myelin mapping has shown promise as an in vivo index of myelin content and may prove useful in testing for altered rates of change in ASD. Noninvasive MRI measures of myelin have been directly validated using myelin stains in marmoset monkeys (Bock et al., 2009) as well as against histological stains in postmortem human brain samples showing correspondence between cyto‐ and myeloarchitecture (Geyer et al., 2011). The T1w/T2w ratio method used in the current study in particular has been demonstrated to be informative across the full range of myelination densities in the cortex, from highly myelinated primary auditory and visual cortices to the more lightly myelinated higher order areas such as anterior insula and cingulate cortex (Glasser et al., 2014). The current study sought to determine if T1w/T2w ratio and its age‐related change trajectories differ between individuals with ASD and age‐matched typical comparison (TC) participants aged 40–70 years. Given substantial heterogeneity in both etiology and outcomes in ASD (Jeste & Geschwind, 2014; Lenroot & Yeung, 2013), the current study also employed subject‐level analyses to examine directional and spatial variability across individuals in the regions demonstrating atypical T1w/T2w ratio. We also expected to find main effect of age, namely, a pattern of cortical demyelination regardless of the diagnostic group, given the findings in typical aging (Peters, 2002; Safaiyan et al., 2016), but with potential accelerated age‐related decline in T1w/T2w ratio in ASD. Finally, we examined links between T1w/T2w ratio and neuropsychological functioning, hypothesizing associations between atypical T1w/T2w ratio and poorer neuropsychological performance in adults with ASD.

## 2 METHODS

### 2.1 Participants

A total of 110 adults (64 with suspected diagnosis of ASD and 46 TC; 40–70 years old) were initially recruited as part of an ongoing longitudinal study of aging in autism. The study was approved by the San Diego State University and University of California, San Diego institutional review boards. All participants (or their conservators) gave written informed consent before participation. Potential participants for the ASD group were recruited through referrals from autism clinics and service organizations, advertisement at local autism‐related events (resource fairs, fund raisers, etc.), and community advertisement. The minimum age of 40 years was selected to ensure that we fully captured the period when neurobiological changes (e.g., parenchymal volume loss and expansion of ventricles) (Longstreth & Cardiovascular Health Study Collaborative Research Group, 1998; Madsen et al., 2015; Schippling et al., 2017) are known to accelerate during typical aging, while the maximum of 70 years was pragmatic, with little chance of recruiting individuals born prior to Kanner's 1943 and Asperger's 1944 first descriptions of the disorder. Adults in the target age range were born between 1945 and 1975. Our understanding of the symptomology of autism has changed substantially over the course of their lifetimes with a steady broadening of diagnostic criteria (American Psychiatric Association, 1952, 2013, 1968, 1980, 1994, 2000). We, therefore, cast a broad net for recruitment of prospective participants for the ASD group, and a preexisting diagnosis of ASD was not required for initial recruitment, but all diagnoses were confirmed as described below before full study inclusion. Participants in the TC group were recruited through community advertisement. Participants with a history of neurological (e.g., epilepsy and tuberous sclerosis) or genetic (e.g., fragile X and Rett syndrome) conditions other than ASD were excluded. TC participants had no family history of autism nor personal history of other neurological conditions or serious mental illness. ASD diagnoses were established by a clinical psychologist based on the Diagnostic and Statistical Manual of Mental Disorders, fifth edition (DSM‐5) criteria and supported by Module 4 of the Autism Diagnostic Observation Schedule, Second Edition (ADOS) (American Psychiatric Association, 2000), along with developmental history when available. Twenty‐seven individuals from the prospective ASD group did not meet these diagnostic criteria and were excluded from further study.

General cognitive abilities were assessed in all participants using the Wechsler Abbreviated Scale of Intelligence, second edition (WASI‐II) (Wechsler, 2011). Aspects of processing speed and executive function were objectively assessed using the Trail Making and Verbal Fluency Tests from the Delis‐Kaplan Executive Function System (DKEFS) (Delis et al., 2001). Specifically, the Number Sequencing and Letter Sequencing conditions from the Trail Making Test were interpreted as processing speed indices, while the Number Letter Sequencing condition reflected set shifting ability. Subjective perception of dysexecutive symptoms was assessed with the Behavior Rating Inventory of Executive Function‐Adult (BRIEF) (Lord et al., 2012), an informant‐report measure, in a subset of participants with an available informant (21 ASD and 18 TC).

### 2.2 MRI data acquisition

Anatomical MRI scans were obtained using a 3 Tesla GE Discovery MR750 scanner with a 32‐channel head coil with built‐in PURE bias correction, and included a T1‐weighted (T1w) magnetization prepared rapid gradient echo (MPRAGE) sequence (Repetition Time = 8.776 ms, Echo Time = 3.656 ms, flip angle = 8°, matrix = 320 × 320, 0.8 mm resolution, 208 slices, slice thickness = 0.8 mm, 8:20 acquisition time; American Psychiatric Association, 2013) and T2‐weighted (T2w) CUBE sequence (TR = 61.803 ms, TE = 3200 ms, flip angle = 8°, matrix = 320 × 320, 0.8 mm resolution, 208 slices, slice thickness = 0.8 mm; 6:51 acquisition time; American Psychiatric Association, 2013). Data included in these analyses are available through the National Institute of Mental Health Data Archive (nda.nih.gov). Qualified researchers may request access through this system.

### 2.3 MRI data processing and quality assessment

Anatomical images were registered in preprocessing using affine boundary based cross‐modal registration (Jenkinson et al., 2012). The T1w images were then processed using FreeSurfer version 5.3.0‐HCP to perform semiautomated cortical reconstruction (Dale et al., 1999; Fischl et al., 1999). Briefly, this processing stream includes removal of non‐brain tissue using a hybrid watershed/surface deformation procedure (Ségonne et al., 2004), automated Talairach transformation, intensity normalization (Sled et al., 1998), tessellation of the gray matter/white matter boundary, automated topology correction (Fischl et al., 2001; Segonne et al., 2007), and surface deformation following intensity gradients to optimally place the gray/white and gray/cerebrospinal fluid borders at the location where the greatest shift in intensity defines the transition to the other tissue class (Dale & Sereno, 1993; Fischl & Dale, 2000). Diagnostic group was masked during MRI data processing and quality assessment. All FreeSurfer output was examined on a slice‐by‐slice basis to identify any inaccuracies in surface placement. Scans with major artifacts, such as ghosting or ringing, or surface placement inaccuracies were excluded. Following visual assessment of anatomical MRI data and FreeSurfer output, data from 10 participants (four ASD and six TC) were excluded from anatomical analyses due to insufficient quality of raw images or surface reconstruction. Data from two additional ASD participants were excluded secondary to atypical neuroanatomical findings (callosal dysgenesis; temporal lobe cyst).

Intracortical MC was estimated in postprocessing by dividing the T1w image by the T2w image and mapping values onto the FreeSurfer‐generated cortical surface in order to facilitate surface‐based analyses (Glasser et al., 2014, 2013, 2014; Glasser & van Essen, 2011). T1w/T2w ratio maps were further quality assessed by visual inspection to ensure proper translation from the cortical ribbon into surface space.

### 2.4 Statistical approach

Group‐level statistical analyses were conducted using a general linear model approach applied on a vertex‐wise basis, testing for a group by age interaction on T1w/T2w ratio as well as main effects of group and age while controlling for non‐verbal IQ (WASI‐II Perceptual Reasoning Index [PRI]), total brain volume (TBV), and gray‐white contrast to noise ratio (CNR) of the T1 images. Gender and its interaction with diagnosis were also tested as variables in the group‐level general linear model, but were removed to conserve degrees of freedom following no significant observed effects. Family wise error correction for multiple comparisons using Permutation Analysis of Linear Models software was implemented (Winkler et al., 2014).

Subject‐level analyses were conducted using an outlier mapping approach. For each individual with ASD, vertex‐wise *z*‐scores were calculated based on the mean and standard deviations of T1w/T2w ratio within the TC group (Marquand et al., 2016). Z‐maps for TC participants were calculated using a leave‐one‐out procedure. Maps were smoothed at a full width at half maximum of 10 mm. A |*z*| ≥ 2 threshold was applied, and maps were cluster‐thresholded to identify “outlier” clusters for each participant. Subsequently, the total surface area of outlier clusters was summed for high T1w/T2w ratio (greater than TC average) and low T1w/T2w ratio (less than TC average) clusters separately, resulting in outlier “load” scores (HR‐load and LR‐load, respectively). Overall group comparisons for T1w/T2w ratio load scores were made using nonparametric Mann–Whitney *U* tests to account for non‐normal distribution of load scores. Load scores were observed to be zero‐inflated across groups, so participants in both diagnostic groups were divided into subgroups based on outlier status. When categorized based on HR‐load, the groups were those with elevated T1w/T2w ratio (ASD‐HR+, *n* = 20; TC‐HR+, *n* = 22) and those without (ASD‐HR−, *n* = 10;TC‐HR−, *n* = 14) and when derived from LR‐load they were those with reduced T1w/T2w ratio (ASD‐LR+, *n* = 9; TC‐LR+, *n* = 13) and those without (ASD‐LR−, *n* = 21; TC‐LR−, *n* = 23).

To examine whether the presence of outliers was associated with diagnosis of ASD, *t*‐tests were used to compare between subgroups based on diagnostic and outlier status, namely, (i) ASD‐HR+ and ASD‐HR− were compared to examine the impact of outlier status within ASD, (ii) ASD‐HR+ and TC‐HR− were compared to examine the impact of both ASD and outlier status, (iii) ASD‐HR+ and TC‐HR+ were compared to examine the impact of diagnostic status within outlier groups, and (iv) TC‐HR+ and TC‐HR− were compared to examine the impact of positive outlier status independent of ASD diagnosis. Corresponding comparisons were performed for LR derived subgroups. Associations between load scores and neuropsychological variables within the HR+ and LR+ groups specifically were tested with Spearman correlations. Behavioral analyses were adjusted for multiple comparisons with the false discovery rate (Benjamini & Hochberg, 1995).

## 3 RESULTS

### 3.1 Group‐level analyses

After completion of quality control steps, anatomical MRI data for 66 participants (30 ASD and 36 TC) were included in subsequent statistical analyses (see Table 1 for demographic characteristics of the final cohort). This included cross‐sectional data collected between 2015 and early 2020. Participants in the ASD and TC groups did not differ significantly in terms of age, nonverbal IQ (WASI‐II PRI), gray‐white CNR, sex, race, or ethnicity. No significant group by age interaction effects were observed on T1w/T2w ratio in the vertex‐wise models. Significant positive main effects of age were observed bilaterally, reflecting increasing T1w/T2w ratio with age broadly across much of the cortex (Table S1), with peak values and largest clusters located in left superior frontal, middle frontal, paracentral, right precentral, superior frontal, and postcentral regions (Figure S1a). Effect sizes for these cross‐sectional age effects were in the moderate range for the largest clusters in each hemisphere (left superior frontal gyrus: *R* ^2 ^= 0.12; right precentral gyrus: 0.17; Figure S1b). Additional smaller clusters were found in frontal, parietal, and temporal lobes as well as the insula, in both hemispheres. There were no significant main effects of group on T1w/T2w ratio in either direction.

**TABLE 1:** Overall participant characteristics and group matching.

	ASD (*n* = 30)			TC (*n* = 36)			
	Mean	SD	Range	Mean	SD	Range	*p*‐value
Age (years)	50.42	6.56	(40.22–67.17)	51.76	7.54	(40.05–69.89)	.452
WASI‐II							
VCI	103.83	22.43	(45–160)	112.47	14.20	(85–144)	.062
PRI	106.74	19.65	(56–138)	109.64	13.27	(75–138)	.768
FSIQ‐4	104.97	20.48	(51–143)	112.56	12.53	(90–138)	.163
Total brain volume	1100.57	105.52	(876–1397)	1131.32	95.95	(886–1309)	.332
Gray‐white CNR	1.39	0.33	(0.57–1.81)	1.57	0.32	(0.46–1.89)	.786
ADOS‐2							
Total score	14.03	4.18	(7–23)	–	–	–	–
SA	10.47	3.78	(5–19)	–	–	–	–
RRB	3.57	1.77	(1–8)	–	–	–	–
Gender (male/female)	23/7			28/			0.758
Race (White/non‐White)	24/4			32/3			0.485
Ethnicity (not Hispanic/Hispanic)	24/3			29/3			0.866

Abbreviations: ADOS‐2, Autism Diagnostic Observation Schedule, Second Edition; ASD, autism spectrum disorder; CNR, contrast to noise ratio; FSIQ, full‐scale IQ; PRI, perceptual reasoning index; RRB, restricted and repetitive behavior; SA, social affect; SD, standard deviation; TC, typical comparison; VCI, verbal comprehension index; WASI‐II, Wechsler Abbreviated Scale of Intelligence, Second Edition.

*p*‐value corresponds to *t*‐test or *χ* ^2^test.

### 3.2 Subject‐level analyses

**FIGURE 1:**
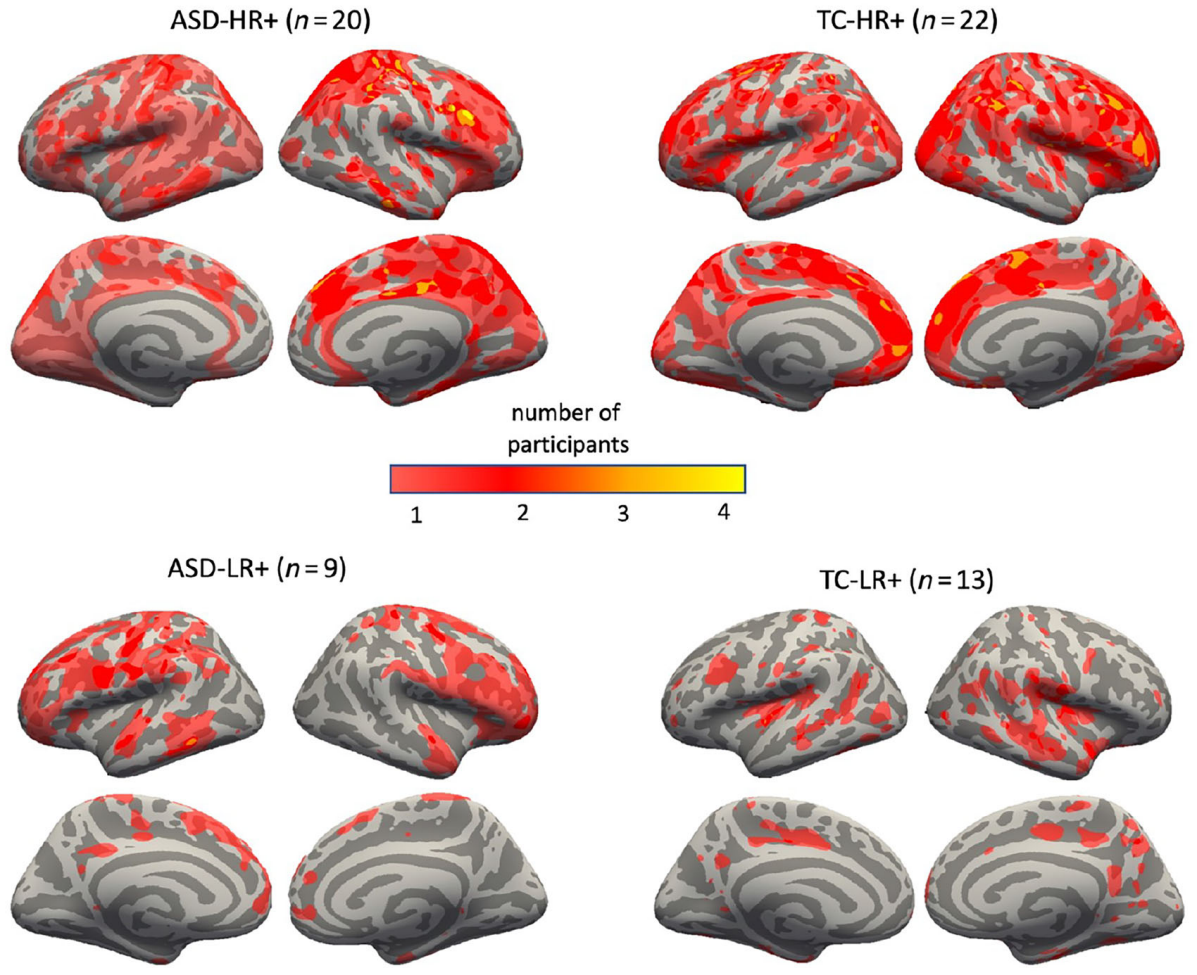
Spatial overlap map of outlier clusters across HR+ and LR+ participants by group. Qualitatively, HR outliers were more abundant than LR outliers in both groups. Both types of outliers were diffused across the cortex and only partially overlapping across participants. ASD, autism spectrum disorder; TC, typical comparison. HR, high T1w/T2w ratio; LR, low T1w/T2w ratio.

Nonparametric *U*‐tests revealed no significant differences between the ASD and TC groups in the distribution of whole‐brain HR or LR outlier load scores. Diagnostic groups were then split by outlier status to account for zero‐inflated data. Subgroups did not differ significantly in terms of age, TBV, or gray‐white CNR (Tables S2 and S3), with the exception of a significant difference in TBV between the ASD‐LR+ and TC‐LR+ groups. Qualitatively, HR outliers were more abundant than LR outliers in both groups (Figure 1). Both types of outliers were diffused across the cortex and only partially overlapping across participants.

**FIGURE 2:**
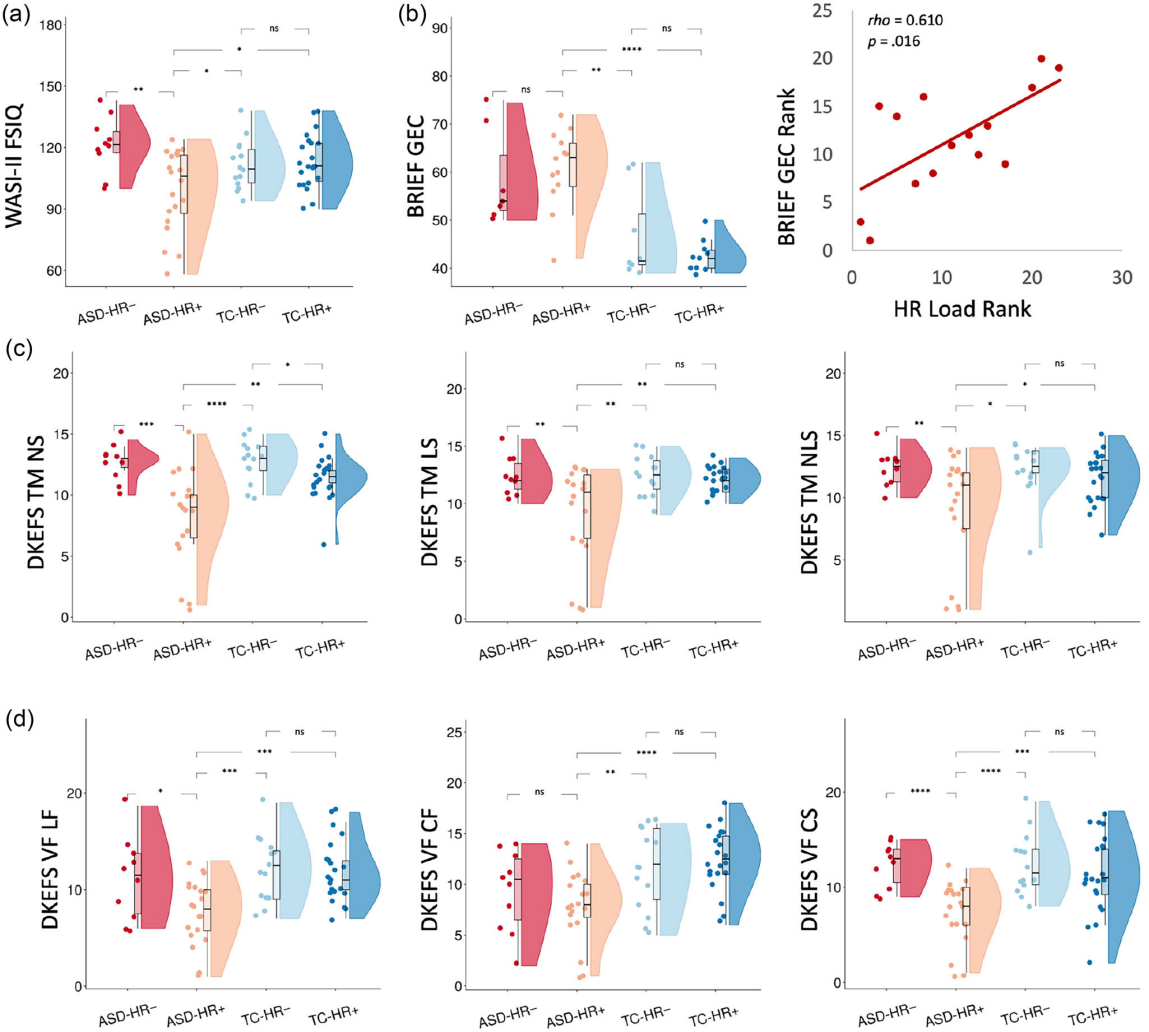
Subgroup comparisons based on HR (high T1w/T2w ratio) status. (a) When groups were split by HR outlier status, the autism spectrum disorder (ASD)‐HR+ group demonstrated significantly lower full‐scale IQ (FSIQ) scores than both the ASD‐HR− and typical comparison (TC)‐HR+ groups. (b) The ASD‐HR+ group showed significantly higher BRIEF GEC (BRIEF is Behavior Rating Inventory of Executive Function‐Adult global executive composite) scores than the TC‐HR+ group, and within the ASD‐HR+ group, BRIEF GEC scores were strongly positively correlated with HR‐load scores, although this association did not remain significant after False Disovery Rate adjustment of *p*‐values. (c) The ASD‐HR+ group showed significantly lower DKEFS (Delis Kaplan Executive Function System) Trail Making Number and Letter Sequencing scores than all other subgroups, and lower Number‐Letter Sequencing scores and both ASD‐HR− and TC‐HR− subgroups. (d) The ASD‐HR+ group showed significantly lower DKEFS Verbal Fluency Letter Fluency and Category Fluency scores than both the TC‐HR− and TC‐HR+ subgroups, along with lower Category Switching scores than all other subgroups. ns, non‐significant; *p < .05; **p < .01; ***p < .001; ****p < .0001.

Results of subgroup comparisons for neuropsychological measures are summarized in Table 2 and Figure 2. The following comparisons were significant following False Discovery Rate adjustment of *p*‐values. When groups were split by HR outlier status, the ASD‐HR+ group demonstrated significantly lower full‐scale IQ scores than both the ASD‐HR− and TC‐HR+ groups. The ASD‐HR+ group showed significantly higher BRIEF GEC (global executive composite) scores than the TC‐HR+ group, and within the ASD‐HR+ group, BRIEF GEC scores were strongly positively correlated with HR‐load scores (*ρ *= 0.610; *p *= .016), although this association did not remain significant after FDR adjustment of *p*‐values. The ASD‐HR+ group showed significantly lower DKEFS Trail Making Number and Letter Sequencing scores than all other subgroups, and lower Number‐Letter Sequencing scores and both ASD‐HR− and TC‐HR− subgroups. The ASD‐HR+ group showed significantly lower DKEFS Verbal Fluency Letter Fluency and Category Fluency scores than both the TC‐HR− and TC‐HR+ subgroups, along with lower Category Switching scores than all other subgroups. The ASD‐HR+ and ASD‐HR− groups showed no significant differences in ADOS scores. All other Spearman correlations within the outlier positive subgroups were nonsignificant.

**TABLE 2:** Subgroup behavioral comparisons (*t*‐test results, *p*‐values).

	High T1w/T2w ratio (HR)			
	ASD‐HR+ vs. ASD‐HR−	ASD‐HR+ vs. TC‐HR−	ASD‐HR+ vs. TC‐HR+	TC‐HR+ vs. TC‐HR−
WASI‐II FSIQ	**0.0014**	0.033	**0.013**	0.71
BRIEF GEC	0.62	0.0039	**0.0000012**	0.27
DKEFS TM NS	**0.00014**	**0.000099**	**0.003**	**0.023**
DKEFS TM LS	**0.01**	**0.006**	**0.009**	0.48
DKEFS TM NLS	**0.008**	**0.012**	0.035	0.35
DKEFS VF LF	0.042	**0.00089**	**0.00023**	0.84
DKEFS VF CF	0.3	**0.0067**	**0.000038**	0.52
DKEFS VF CS	**0.000055**	**0.000072**	**0.00099**	0.37
	**Low T1w/T2w ratio (LR)**			
	ASD‐LR+ vs. ASD‐LR−	ASD‐LR+ vs. TC‐LR−	ASD‐LR+ vs. TC‐LR+	TC‐LR+ vs. TC‐LR−
WASI‐II FSIQ	0.35	0.72	0.41	0.07
BRIEF GEC	0.94	0.014	0.048	0.55
DKEFS TM NS	0.6	0.3	0.24	0.72
DKEFS TM LS	0.98	0.26	0.34	0.54
DKEFS TM NLS	0.72	0.47	0.41	0.79
DKEFS VF LF	0.67	0.11	0.015	0.18
DKEFS VF CF	0.49	0.0066	0.0037	0.37
DKEFS VF CS	0.26	0.2	0.45	0.49

*Note*: Bold *p*‐values remain significant after FDR adjustment.

Abbreviations: ASD, autism spectrum disorder; BRIEF, Behavior Rating Inventory or Executive Function; CF, category fluency; CS, category switching; DKEFS, Delis Kaplan Executive Function System; FSIQ, full‐scale IQ; GEC, global executive composite; LF, letter fluency; LS, letter sequencing; NLS, number letter sequencing; NS, number sequencing; TC, typical comparison; TM, trail making; VF, verbal fluency; WASI‐II, Wechsler Abbreviated Scale of Intelligence, Second Edition.

When groups were split by LR outlier status, *t*‐test comparisons revealed no significant differences between subgroups after FDR adjustment of *p*‐values. Similarly, Spearman correlations within the LR+ subgroups were nonsignificant.

## 4 DISCUSSION

The present study used group‐ and subject‐level approaches to examine intracortical T1w/T2w ratio, an index of myelin content, and its associations with age and neuropsychological function in middle to older aged adults with ASD compared to TC participants. Vertex‐wise analyses at the group level revealed a positive main effect of age on T1w/T2w ratio, with greater age was associated with higher T1w/T2w ratio in clusters across bilateral frontal, parietal, and temporal lobes. In subject‐level analyses, diagnostic groups did not differ in terms of overall outlier load, but after accounting for zero‐inflation of load scores by subgrouping participants based on load status, associations between elevated outlier load and various aspects of neuropsychological function were detected. The presence of atypically high T1w/T2w ratio at the subject‐level was associated with a number of differences in neuropsychological function, with poorer performance on average in the ASD subgroup with atypically high T1w/T2w ratio in spatially heterogeneous cortical regions. Differences were noted across several cognitive domains, including overall intellectual functioning, processing speed, and various aspects of executive function.

### 4.1 Clusters of atypically high T1w/T2w ratio in both groups

Our subject‐level approach revealed outlier clusters of high T1w/T2w ratio in many participants in both the ASD and TC groups, which varied in their exact regional localization from person to person. High regional T1w/T2w ratio could reflect developmental differences (local or diffuse) in the rate of ongoing myelination into adulthood. However, it should be noted that the total area of outlier clusters did not differ between diagnostic groups, indicating that patches of HR are not specific to middle to older aged adults with ASD. Given evidence suggesting that an important role of intracortical myelin is to insulate against formation of aberrant connections (Braitenberg, 1962), these HR clusters could underly individual differences in cortical connectivity (Hensch, 2005; Hill et al., 2018; McGee et al., 2005; Nave & Werner, 2014). The specific circuits and networks impacted by T1w/T2w ratio differences could potentially be associated with different neurocognitive phenotypes. Although comparison of overall outlier load scores did not reveal differences between the ASD and TC groups as hypothesized, subgrouping participants based on HR load status revealed associations with neuropsychological functions, indicating a potential link between altered T1w/T2w ratio and neurocognitive profiles in ASD. The individual level spatial distributions of HR+ clusters could reflect genetic or early neurodevelopmental differences, and although not directly related to diagnostic status, it may be associated with neurobehavioral differences present earlier in life (Ecker et al., 2022). Alternatively, HR clusters could reflect a compensatory response to aging‐related functional decline, with added myelin forming to guide the development of new connections given what has been shown about myelin's role in nervous system plasticity (Bonetto et al., 2021; Fields, 2015; Forbes & Gallo, 2017).

Within both diagnostic groups, the spatial distribution of both HR and LR clusters was highly variable between subjects. Although our sample size was not sufficient to quantitatively test for areas of high susceptibility (Bethlehem et al., 2020), visual inspection of the overlap of HR clusters in our sample suggested a more anterior (frontal and parietal) distribution of HR in the ASD group than in the TC group (Figure 1). It is likely that spatial variability in atypical myelin content reflects another aspect of the heterogeneity that characterizes ASD at multiple levels. Along with heterogeneity in the symptom profile and severity (Esbensen et al., 2009; Estes et al., 2007; Pickles et al., 2014), risk and prognostic factors are variable and complex, and the genetic landscape of ASD is vast. A number of genetic variants have been shown to confer increased risk of ASD (Pinto et al., 2010; Yuen et al., 2017). Additionally, older aged adults with ASD are subject to a lifetime of environmental (Garay & McAllister, 2010; Larsson et al., 2005; Mandy & Lai, 2016; Patel et al., 2018; Vargas et al., 2005), experiential, interventional (Helt et al., 2008; Klintwall et al., 2015; Wong et al., 2015), and pharmacological (Broadstock et al., 2007; Howes et al., 2018) contributions to their neurobehavioral and neurocognitive outcomes. Co‐occurring physical and mental health conditions are also common across the lifespan in ASD (Dhanasekara et al., 2023; Hand et al., 2020), with certain conditions becoming more prevalent with increasing age (Fortuna et al., 2016). Together, highly heterogeneous etiologies in ASD that are further compounded by other sources of variability across the lifespan have the potential to differentially impact brain structure and neural resilience later in life.

### 4.2 Relationships between atypical T1w/T2w ratio and neuropsychological functions in ASD

After splitting each diagnostic group based on HR load status, the ASD‐HR+ group showed several significant differences from all other groups. When compared to the ASD‐HR− group, the ASD‐HR+ group showed lower IQ and poorer processing speed and set‐shifting ability, along with a positive association between load scores and overall dysexecutive symptoms. This is notable considering inconsistency in previous studies of neuropsychological profiles of ASD across the adult lifespan, with some studies reporting deficits in intellectual, executive, and attentional functioning (Brighenti et al., 2018; Fried et al., 2016), among other cognitive domains (Lever & Geurts, 2016; Torenvliet et al., 2023), and others demonstrating no significant differences compared to typical comparison groups (Brighenti et al., 2018; Geurts et al., 2020; Torenvliet et al., 2023; Wilson et al., 2014). Subgrouping or clustering of individuals with ASD based on a variety of features, including neuroimaging characteristics (Hong et al., 2020; Katuwal et al., 2016) and other medical or genetic factors (Ousley & Cermak, 2014), has been broadly utilized as a means of parsing heterogeneity in the disorder (Ousley & Cermak, 2014). The present results suggest that subgrouping by HR load status may have utility in exploring links between brain structure and behavior despite intersubject variability.

When comparing the ASD‐HR+ group to the TC subgroups, ASD‐HR+ participants showed lower performance on all tests included. In contrast, performance in the ASD‐HR− group was similar to that in both the TC‐HR− and TC‐HR+ groups across most tests, and the TC subgroups did not show differences from one another based on HR outlier status. However, the distribution of scores in the TC group was narrow, potentially limiting our ability to detect similar effects. While this may reflect a true difference in the functional impact of high versus low myelin content, it is also possible that the relatively small number of participants in the ASD‐LR+ (*n* = 9) and TC‐LR+ (*n* = 13) groups limited the statistical power to detect associations with neuropsychological function.

### 4.3 Age related increases in MC

The present study showed a main effect of linearly increasing T1w/T2w ratio with age in group‐level analyses, with no evidence for any group difference in the age‐related trajectory of intracortical T1w/T2w ratio. Linear increases in cortical myelin content have been shown at younger ages extending into at least the fourth decade of life. (Shafee et al., 2015) More recently, a significant linear increase with age was found across most of the frontal lobe and in temporo‐parietal regions in typically aging participants in the same age range as the current study (Parent et al., 2023). Correlations with gene expression data in the same study suggest that this increase may be related to oligodendrocyte and oligodendrocyte precursor cell density (Seidlitz et al., 2020). Thus, age‐related increases in the T1w/T2w ratio could potentially be driven by increased myelination; by relative decreases in neurons, neurites, or other glial cell types; or by a combination of these. The primary microstructural drivers of the MRI‐based measures could also vary by region, age, or individual (Parent et al., 2023).

Other studies employing different MC estimation methods and nonlinear models suggest that a linear model may not provide the best fit. MC indexed by myelin water fraction (MWF) showed an inverted U‐shaped relationship across most cerebral white and gray matter (Dvorak et al., 2021), although this approach was limited by the use of a single mask averaged across all gray matter, potentially failing to account for regional variability in the age‐related trajectory of cortical MC. The same pattern has also been demonstrated in a study looking specifically at MWF in individual subcortical gray matter structures (Khattar et al., 2021). It was also noted that these quadratic relationships were driven largely by study participants <25 years of age, and a study spanning young adulthood has shown similar patterns with earlier peak ages (Rowley et al., 2017). It is likely that cortical gray matter myelination follows a different maturational pattern, depending heavily on the age range sampled. Sample size limited our ability to test a quadratic relationship, and further investigation in larger sample sizes and with longitudinal methods will be necessary to establish these trajectories.

### 4.4 Limitations and conclusions

Although this is one of few studies examining brain structure in middle to older aged adults with ASD, and to our knowledge the first to examine T1w/T2w ratio in such groups, some limitations must be acknowledged when interpreting these results. First, the T1w/T2w ratio method employed here provides a proxy index of myelin content and may be subject to influence from other aspects of cortical brain anatomy, including iron content (Fukunaga et al., 2010), or to errors in surface reconstruction impacting the precision of its estimate. Other methods of measuring myelin content using MRI include MWF, magnetization transfer ratio, and relaxometry, all of which have shown high correlations with histology (Mancini et al., 2020; van der Weijden et al., 2021). In contrast with the positive main effect of age on T1w/T2w observed here, there have been previous reports of negative age effects when using MWF, but many of those studies focus on white matter structures or the basal ganglia and thalamus (Billiet et al., 2015; Bouhrara et al., 2020; Faizy et al., 2020). Findings in the cortex however are mixed. For example, one study that measured MWF separately in cerebral cortex, cerebral white matter, and cerebral deep gray matter in 20‐ to 80‐year‐olds showed substantial MWF decline in white matter and deep gray matter but minimal difference in the 40–70 year range in cortex (Zhou et al., 2023). Notably, the T1w/T2w ratio method also has important advantages when compared to similar MRI based metrics of MC, including elimination of the MR‐related image intensity bias and enhancement of the contrast to noise ratio for myelin. The method is informative across the full range of cortical myelin densities and demonstrates good correspondence with myeloarchitectonic maps from animal and postmortem studies (Glasser & van Essen, 2011).

Second, the relatively modest sample size and highly exploratory nature of the subject‐level analyses likely limit the generalizability of these results. In particular, our sample size did not allow for inquiries into differences in the specific spatial localization of outlier clusters between groups on a lobar or region wise level, and this research question would benefit from follow up with a larger sample. Finally, the cross‐sectional approach limits interpretation of the findings with regard to differentiating between effects arising early in development, compensatory changes occurring across the lifespan, and neurodegenerative changes in later life. Longitudinal studies will be necessary to elucidate these factors and are currently underway.

The protracted nature of myelination in humans makes it unique as an index of brain development and degeneration, making it vital to study in developmental disorders from a lifespan perspective. The multifaceted group and subject‐level approaches employed herein demonstrate the importance of accounting for increasing sources of heterogeneity when studying older adults with ASD. Despite its limitations, the current study provides important preliminary insights into relationships between brain structure and cognition in the second half of the lifespan in ASD, suggesting shared factors contributing to atypical intracortical myelin content and poorer cognitive outcomes for a subset of middle aged to older autistic adults. These atypicalities likely reflect diverse histories of neurodevelopmental deficits, and possible compensatory changes, compounded by processes of aging, and may serve as useful markers of vulnerability to further cognitive decline in older adults with ASD. Our findings add to a limited but vital literature on the neurobiological and cognitive changes that occur during aging in ASD.

## AUTHOR CONTRIBUTIONS

**Jiwandeep S. Kohli**: Conceptualization; data curation; formal analysis; investigation; methodology; project administration; visualization; writing—original draft; writing—review and editing. **Annika C. Linke**: Conceptualization; methodology; writing—original draft; writing—review and editing. **Ian A. Martindale**: Data curation; project administration; writing—review and editing. **Molly Wilkinson**: Data curation; project administration; writing—review and editing. **Mikaela K. Kinnear**: Data curation; project administration; supervision; writing—review and editing. **Alan J. Lincoln**: Conceptualization; supervision; writing—review and editing. **Janice Hau**: Data curation; writing—review and editing. **Ian Shryock**: Data curation; project administration; writing—review and editing. **Vinton Omaleki**: Project administration; resources; writing—review and editing. **Kalekirstos Alemu**: Data curation; project administration; writing—review and editing. **Stephanie Pedrahita**: Data curation; project administration; writing—review and editing. **Inna Fishman**: Project administration; supervision; writing—review and editing. **Ralph‐Axel Muller**: Conceptualization; funding acquisition; supervision; writing—review and editing. **Ruth A. Carper**: Conceptualization; data curation; funding acquisition; project administration; supervision; writing—original draft.

## CONFLICT OF INTEREST STATEMENT

The authors declare no conflicts of interest.

### PEER REVIEW

The peer review history for this article is available at https://publons.com/publon/10.1002/brb3.3594

## Supporting information

Supplementary Table 1 Clusters of greater T1w/T2w ratio with older age
Supplementary Table 2 Subgroup Characteristics
Supplementary Table 3 Subgroup Matching (p‐values)
Supplementary Figure 1 Main effect of age on T1w/T2w ratio

## Data Availability

Data from the current study are available through the National Institute of Mental Health Data Archive (nda.nih.gov), reference number R01MH103494. Qualified researchers may request access through this system.
