# Supplementary material for: Associations between atypical intracortical myelin content and neuropsychological functions in middle to older aged adults with ASD
Source: Brain Behav. 2024 Jun 7;14(6):e3594. doi: 10.1002/brb3.3594 (PMC11161394; doi:10.1002/brb3.3594)
Supplement: Supplementary file 1 — Supplementary Table 1 Clusters of greater T1w/T2w ratio with older age Supplementary Table 2 Subgroup Characteristics Supplementary Table 3 Subgroup Matching (p‐values) Supplementary Figure 1 Main effect of age on T1w/T2w ratio [file BRB3-14-e3594-s001.docx]

| **Supplementary Table 1** Clusters of greater T1w/T2w ratio with older age | | | | | | |
| --- | --- | --- | --- | --- | --- | --- |
| **Hemisphere** | **Location** | **Cluster-Wise p-value** | **Size (mm^2^)** | **MNI-X** | **MNI-Y** | **MNI-Z** |
| Left | superiorfrontal | 0.0002 | 900.76 | -9.8 | 1.2 | 49.5 |
|  | rostralmiddlefrontal | 0.0002 | 825.55 | -18.4 | 58.3 | -13.7 |
|  | paracentral | 0.0002 | 438.05 | -7.9 | -26.5 | 51.5 |
|  | parsopercularis | 0.0002 | 421.05 | -46.9 | 12.7 | 3 |
|  | lateralorbitofrontal | 0.0002 | 367.95 | -26.7 | 23.7 | -4.4 |
|  | precentral | 0.0002 | 267.79 | -40 | 1 | 27.1 |
|  | insula | 0.0002 | 264.74 | -37.7 | -16.5 | 20.9 |
|  | transversetemporal | 0.0002 | 245 | -44.8 | -22.8 | 7.6 |
|  | superiortemporal | 0.0002 | 226.21 | -49.6 | -36.5 | 9.1 |
|  | precentral | 0.0002 | 223.88 | -32.4 | -8.4 | 45 |
|  | postcentral | 0.0008 | 177.94 | -48.8 | -17.9 | 35 |
|  | superiorfrontal | 0.00519 | 145.92 | -19.4 | 9.1 | 55.9 |
|  | precentral | 0.00619 | 141.69 | -52.3 | 3.7 | 10.7 |
|  | insula | 0.01097 | 133.21 | -35.5 | -3.5 | 10.1 |
|  | lateralorbitofrontal | 0.01475 | 125.93 | -29.8 | 30.3 | -14.2 |
|  | parsorbitalis | 0.01613 | 124.08 | -38.1 | 42.7 | -11.7 |
| Right | precentral | 0.0002 | 3163.67 | 59.6 | 5.6 | 23.9 |
|  | superiorfrontal | 0.0002 | 1407.81 | 10.2 | 7.1 | 46.9 |
|  | postcentral | 0.0002 | 1088.82 | 60.6 | -11.8 | 31.7 |
|  | middletemporal | 0.0002 | 520.92 | 61 | -10.7 | -23.9 |
|  | superiortemporal | 0.0002 | 466.54 | 66.1 | -25.4 | 4.3 |
|  | superiortemporal | 0.0002 | 369.65 | 52 | -11.1 | -1.3 |
|  | inferiorparietal | 0.0002 | 363.46 | 54 | -52.8 | 35.4 |
|  | superiorparietal | 0.0002 | 342.66 | 29.6 | -48.9 | 46.2 |
|  | caudalmiddlefrontal | 0.0002 | 217.85 | 38.3 | 19.8 | 35.4 |
|  | precentral | 0.0002 | 208.29 | 32 | -17.7 | 44.4 |
|  | superiorfrontal | 0.0008 | 185.69 | 20.7 | -4.9 | 57.9 |
|  | insula | 0.002 | 167.31 | 36 | 9 | -9.3 |
|  | precuneus | 0.002 | 166.32 | 7.1 | -71.4 | 48 |
|  | middletemporal | 0.0022 | 165.28 | 62.1 | -49.8 | 1.6 |
|  | rostralmiddlefrontal | 0.0022 | 164.8 | 29.9 | 49.1 | 2.2 |
|  | inferiorparietal | 0.0022 | 160.07 | 42.7 | -72.4 | 16.4 |
|  | precuneus | 0.00579 | 141.23 | 8.6 | -52.5 | 56 |
|  | superiorparietal | 0.00659 | 136.75 | 20.5 | -86.7 | 29.2 |
|  | supramarginal | 0.03194 | 110.77 | 57.9 | -41 | 27.8 |
|  | caudalmiddlefrontal | 0.04058 | 107.25 | 36.3 | 8.7 | 38 |

| **Supplementary Table 2** Subgroup Characteristics | | | | | | | | | | | | |
| --- | --- | --- | --- | --- | --- | --- | --- | --- | --- | --- | --- | --- |
|  | High T1w/T2w ratio (HR) | | | | | | | | | | | |
|  | ASD-HR- (n=10) | | | ASD-HR+ (n=20) | | | TC-HR- (n=14) | | | TC-HR+ (n=22) | | |
|  | Mean | SD | Range | Mean | SD | Range | Mean | SD | Range | Mean | SD | Range |
| Age (years) | 53.13 | 6.65 | [41.92-67.17] | 49.06 | 6.24 | [40.22-63.66] | 51.63 | 8.53 | [40.54-69.89] | 51.84 | 7.05 | [40.05-64.81] |
| Total Brain Volume | 1101.42 | 71.03 | [1013-1190] | 1109.84 | 120.75 | [876-1397] | 1103.71 | 112.39 | [886-1309] | 1148.88 | 81.80 | [1005-1309] |
| Gray White CNR | 1.32 | 0.40 | [0.65-1.75] | 1.41 | 0.30 | [0.57-1.81] | 1.52 | 0.14 | [1.36-1.89] | 1.33 | 0.37 | [0.45-1.67] |
|  | Low T1w/T2w ratio (LR) | | | | | | | | | | | |
|  | ASD-LR- (n=21) | | | ASD-LR+ (n=9) | | | TC-LR- (n=23) | | | TC-LR+ (n=13) | | |
|  | Mean | SD | Range | Mean | SD | Range | Mean | SD | Range | Mean | SD | Range |
| Age (years) | 51.62 | 6.67 | [403.16-67.17] | 49.29 | 6.30 | [40.22-63.66] | 53.28 | 7.57 | [41.05-69.89] | 49.08 | 6.97 | [40.05-57.94] |
| Total Brain Volume | 1125.85 | 102.76 | [955-1397] | 1110.56 | 114.50 | [876-1397] | 1120.83 | 103.39 | [886-1309] | 1149.87 | 81.67 | [1031-1309] |
| Gray White CNR | 1.38 | 0.34 | [0.62-1.81] | 1.43 | 0.29 | [0.57-1.81] | 1.43 | 0.24 | [0.71-1.67] | 1.37 | 0.43 | [0.45-1.89] |
| ASD, Autism Spectrum Disorder; TC, Typical Comparison; WASI-II, Wechsler Abbreviated Scale of Intelligence, Second Edition; VCI, Verbal Comprehension Index; PRI, Perceptual Reasoning Index; FSIQ, Full-Scale IQ; CNR, Contrast to Noise Ratio; ADOS-2, Autism Diagnostic Observation Schedule, Second Edition; SA, Social Affect; RRB, Restricted and Repetitive Behavior | | | | | | | | | | | | |

| **Supplementary Table 3** Subgroup Matching (p-values) | | | | |
| --- | --- | --- | --- | --- |
| High T1w/T2w ratio (HR) | | | | |
|  | Subgroup | ASD-HR+ | TC-HR- | TC-HR+ |
| Age (years) | ASD-HR- | 0.082 | 0.649 | 0.631 |
|  | ASD-HR+ | -- | 0.316 | 0.184 |
|  | TC-HR- | -- | -- | 0.611 |
|  | TC-HR+ | -- | -- | -- |
| Total Brain Volume (cm^3^) | ASD-HR- | 0.812 | 0.955 | 0.124 |
|  | ASD-HR+ | -- | 0.882 | 0.223 |
|  | TC-HR- | -- | -- | 0.114 |
|  | TC-HR+ | -- | -- | -- |
| Gray White CNR | ASD-HR- | 0.188 | 0.103 | 0.969 |
|  | ASD-HR+ | -- | 0.219 | 0.453 |
|  | TC-HR- | -- | -- | 0.067 |
|  | TC-HR+ | -- | -- | -- |
| Low T1w/T2w ratio (LR) | | | | |
|  | Subgroup | ASD+ | TC- | TC+ |
| Age (years) | ASD-HR- | 0.186 | 0.446 | 0.297 |
|  | ASD-HR+ | -- | 0.517 | 0.607 |
|  | TC-HR- | -- | -- | 0.110 |
|  | TC-HR+ | -- | -- | -- |
| Total Brain Volume (cm^3^) | ASD-HR- | 0.807 | 0.873 | 0.481 |
|  | ASD-HR+ | -- | 0.167 | **0.041** |
|  | TC-HR- | -- | -- | 0.391 |
|  | TC-HR+ | -- | -- | -- |
| Gray White CNR | ASD-HR- | 0.092 | 0.647 | 0.888 |
|  | ASD-HR+ | -- | 0.634 | 0.955 |
|  | TC-HR- | -- | -- | 0.594 |
|  | TC-HR+ | -- | -- | -- |
| ASD, Autism Spectrum Disorder; TC, Typical Comparison; HR, High T1w/T2w ratio; LR, Low T1w/T2w ratio; CNR, Contrast to Noise Ratio  **Bold = significant difference between groups** | | | | |


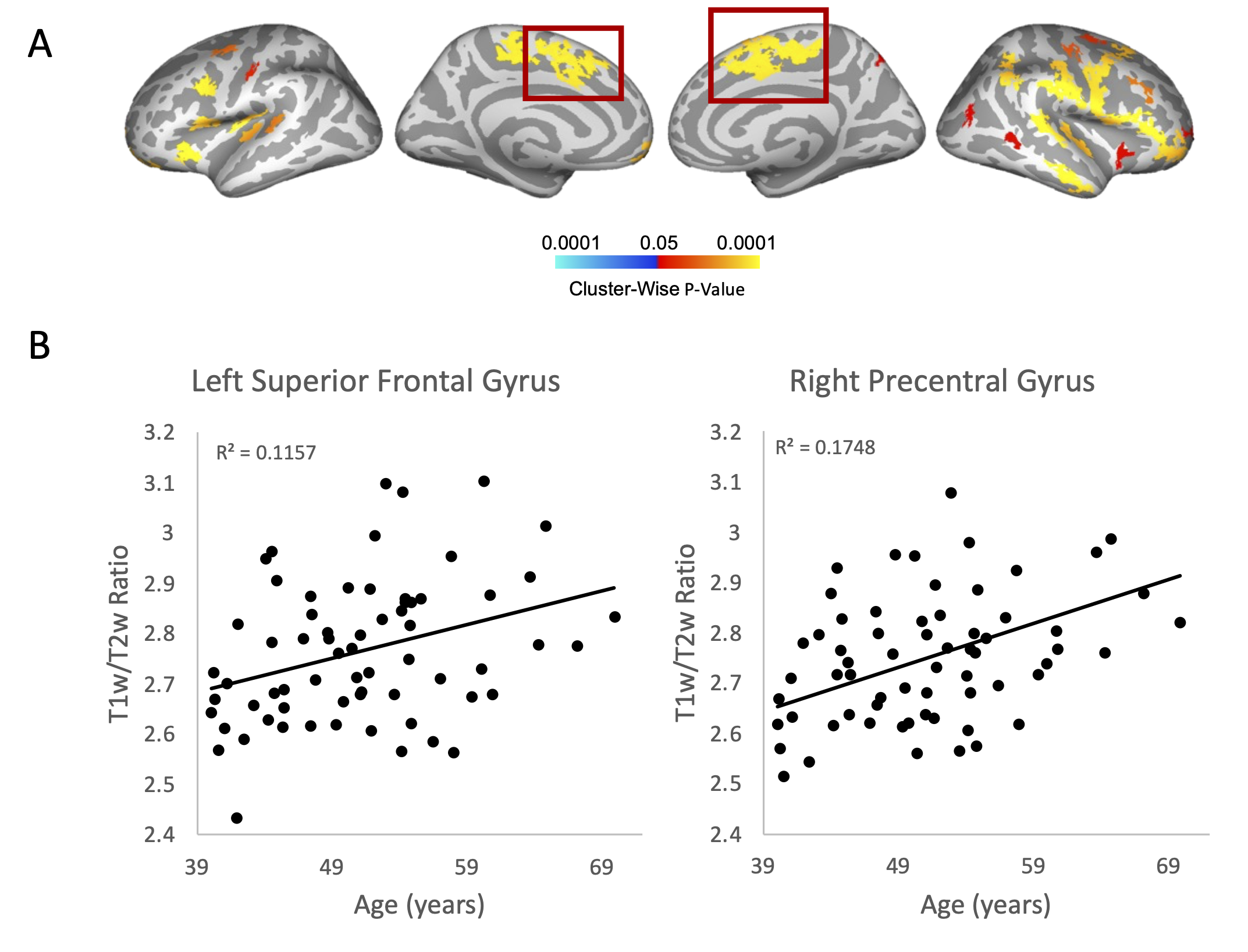


**Supplementary Figure 1 Main effect of age on T1w/T2w ratio**

(A) Significant positive main effects of age were observed bilaterally, reflecting increasing T1w/T2w ratio with age broadly across much of the cortex, with peak values and largest clusters located in left superior frontal, middle frontal, paracentral, and right precentral, superior frontal, and postcentral regions. Additional smaller clusters were found in frontal, parietal, and temporal lobes as well as the insula, in both hemispheres. (B) Exemplary scatterplots for the largest clusters with peak values located on the left superior frontal gyrus and right precentral gyrus, demonstrating the modest positive cross-sectional association between age and T1w/T2w ratio.
